# What Factors Predict the Adoption of Type 2 Diabetes Patients to Wearable Activity Trackers—Application of Diffusion of Innovation Theory

**DOI:** 10.3389/fpubh.2021.773293

**Published:** 2022-01-03

**Authors:** Ping Chen, Ying Shen, Zeming Li, Xinying Sun, Xing Lin Feng, Edwin B. Fisher

**Affiliations:** ^1^Department of Social Medicine and Health Education, School of Public Health, Peking University, Beijing, China; ^2^Global Health Office, Beijing Center for Disease Prevention and Control, Beijing, China; ^3^Department of Health Policy and Management, School of Public Health, Peking University, Beijing, China; ^4^Department of Health Behavior Gillings School of Global Public Health, University of North Carolina at Chapel Hill, Chapel Hill, NC, United States

**Keywords:** physical activity, wearable activity trackers, type 2 diabetes, elderly patients, diffusion of innovation theory, structural equation modeling

## Abstract

**Background:** Globally, diabetes has brought an enormous burden to public health resources, and the situation of disease burden caused by diabetes in China is especially severe. China is currently facing the dual threat of aging and diabetes, and wearable activity trackers could promote elderly diabetic patients' physical activity levels and help them to manage blood glucose control. Therefore, examining the influencing factors of elderly patients' adoption intention is critical as wearing adoption determines actual wearing behaviors.

**Objective:** This study aims to explore the predicting factors of Chinese elderly type 2 diabetic patients' adoption intention to wearable activity trackers and their actual wearing behavior, using diffusion of innovation theory as the theoretical framework. We hope to provide insights into future interventions using wearable activity trackers as tools to improve the outcome of patients.

**Methods:** Wearable activity trackers were freely distributed to type 2 diabetic patients in Beijing, China. A questionnaire survey was conducted to examine predicting factors of adoption intention after a week's try-on. Actual wearing behavior for 3-month was obtained from the exclusive cloud. Data were analyzed with structural equation modeling.

**Results:** A total of 725 patients completed the questionnaire. Patients had a mean age of 60.3 ± 7.6 years old and the educational level was generally lower. The results indicated that observability was the primary influencing factor of patients' adoption intention (β = 0.775, *P* < 0.001). Relative advantage (β = 0.182, *P* = 0.014) and perceived social image (β = 0.080, *P* = 0.039) also had a positive influence while perceived risk (β = −0.148, *P* < 0.001) exerted a negative influence. In addition, results showed that the more intention led to the better actual wearing behavior (β = 0.127, *P* = 0.003). Observability (β = 0.103, *P* = 0.005), perceived ease (β = 0.085, *P* = 0.004), and relative advantage (β = 0.041, *P* = 0.009) also indirectly influenced the wearing behavior.

**Conclusion:** The intentions of Chinese elderly type 2 diabetic patients to wearable activity trackers directly influenced the actual wearing behavior. In addition, their adoption intention to wearable activity trackers was mainly influenced by observability, perceived ease to use, relative advantage, perceived risk, and social image.

## Introduction

### Background

Globally, diabetes has brought an enormous burden to public health resources and from 2006 to 2016, the number of deaths attributed to diabetes has increased by 31.1% (1, 2). According to the statistics reported by International Diabetes Atlas (IDF) in 2017, currently, there is 425 million diabetes patients worldwide and without effective interventions, this number would increase to 629 million by 2045 (3). The situation of diabetes in China is even more overwhelming. It was estimated that the prevalence of diabetes in China had increased from 2.6% in 2002 to 10.4% in 2013, and the trend was continuing (4). Despite the great threat that elevated blood glucose posed to patients' health, only 15.8% had their glucose level under control (4).

Among various lifestyle modifications, physical activity intervention was proven to be effective in improving diabetes patients' blood glucose control. Several meta-analyses compared physical activity intervention to diabetes patients with usual care and the results indicated a decrease of HbA_1C_ between 0.6 and 1.0% (5–8). However, despite the importance of physical activity, patients' participation in exercise is not optimistic. Research has revealed that in China, only 45% of patients with diabetes performed regular exercise and most newly diagnosed patients had inadequate physical activity (9, 10). A nationwide survey recruiting 5,916 diabetes patients reported that only 35.2% of patients stuck to physical activity regimen and 29.3% never participated in any forms of exercise (11). Several reasons caused patients inactivity and according to literature, the most common and important ones are poor self-efficacy, lack of motivation, and lack of monitoring devices (5, 7).

Fortunately, newly emerged technologies such as multifunctioning wearable activity trackers could well address the above barriers to physical activity. Several studies had examined the effectiveness of simple step-recording pedometers in improving diabetes patients' physical activity and the results indicated 2,000~3,000 steps increase (12–19). Different from pedometers, wearable activity trackers are able to offer more behavior change techniques and implement them in different ways as compared with standard displays on the device (17). For example, most activity trackers could connect with mobile applications and form an online community. Through social support and peer pressure, users might have higher motivations to perform physical activity. Furthermore, wearable activity trackers also have the function of real-time heart-rate monitoring and goal setting, which could serve to increase moderate-intensity physical activities and the total activity amount. Miyauchi et al. reported superior efficacy of activity monitors over pedometers in type 2 diabetes patients (20) and other studies exploring the effect of wearables in the general population also reported significant improvements in physical activity levels and related parameters (16, 17, 19, 21).

Although wearable activity trackers are proven to be effective in lowering blood glucose in diabetes patients, their acceptability among those patients is scarcely studied, especially in China (17). Currently, most acceptability studies related to wearable activity trackers were conducted in healthy populations and the sample size was relatively small. An acceptability study conducted by Puri et al. in elderly patients showed that equipment's display, battery life, aesthetics, and comfort had a significant influence on device acceptance and privacy issue was less of a concern. However, this and other elderly studies were mainly conducted in highly educated healthy population in developed countries and the population characteristics are vastly different from the general Chinese elderly population with type 2 diabetes (22, 23). China had 25% of the world's total diabetes patients and the speed of population aging has surpassed the rate of aging for the rest of the world (3, 24). In 2010, the number of Chinese people aged over 60 years exceeded 194 million, accounting for 14.3% of the total population (24). China is facing the dual threat of diabetes and aging and if older diabetes patients in China are able to use new technologies to improve physical activity and ultimately glucose control, the social burden and health system burden could be substantially alleviated (25). Normally, users' acceptance determines usage behavior, and elderly diabetic patients will only accept and adopt wearable activity tracker when it meets their needs and expectations (26). As a result, to ensure that patients could fully enjoy the advantages of wearable activity trackers, the factors influencing their adoption intentions should be first discussed.

### Theoretical Basis

Diffusion of innovation theory (DOI) could depict the fundamental characteristics of innovation and the adoption curves of people through time. From the perspective of describing the characteristics of innovation, DOI could help indicate the influencing factors of adoption intention for innovative things and it has been proven a significant predictor of adoption intention (27, 28). Therefore, in this cross-sectional study, DOI was applied to depict the influencing factors of adopt intention. In the original framework of DOI, relative advantage, observability, compatibility, and trialability were considered to positively influence users' adoption intention while perceived complexity had a negative influence (29).

Relative advantage reflected the degree to which wearable activity trackers were perceived useful by type 2 diabetes patients (29). Li et al. reported that among various innovation characteristics of smart wearables, relative advantage was the primary influencing factor and other studies exploring adoption intention of the smartphone, mHealth, and telehealth also reported a positive influence of relative advantage (24, 30–33).

Observability referred to whether the benefits of a wearable activity tracker were easily observed and visible (29). If patients could easily observe the effects of wearable activity trackers, they were more likely to adopt it for a long time.

Compatibility referred to whether a wearable activity tracker was compatible with patients' values, beliefs, experiences, and needs (29, 34). If the use of wearables was compatible with patients' past experiences and habits, patients' adoption intention might be higher (32).

Perceived complexity reflected the efforts patients demonstrated when trying to adopt wearable activity trackers (29). In this study, patients need to regularly charge the wearable activity trackers and connect it to a smartphone to transmit data. Therefore, the operation complexity might hinder patients' adoption intention (31). Apart from the direct influence, research also proposed that perceived complexity had an indirect effect through relative advantage and observability on adoption intention (24, 30–32).

Trialability referred to the degree to which innovation could be tried by potential users (29). In the current study, as wearable activity trackers were freely distributed to patients, trialability was not applicable thus we only included the remaining four dimensions of DOI.

Through a literature search, we found that apart from the five dimensions of DOI, perceived social image and perceived risk could also influence users' adoption intention to innovation products. First, in this study, patients need to download the Lifesense Application, register an account and transmit physical activity data regularly. Therefore, some patients might worry about personal information leakage and thus are unwilling to adopt wearable activity trackers (30, 32). As a result, we introduced perceived risk to the original DOI framework. Second, wearable activity trackers had the function of accessories and users might adopt an innovative technology to reflect their social status and improve their social image. Venkatesh et al. explored the influencing factors of personal laptop buying behavior and the result indicated that for pioneers, a personal laptop could improve their social image was the most important factor (26). Therefore, we hypothesized that perceived social image could improve patients' adoption intention of wearable activity trackers.

### Theoretical Hypotheses

**Figure 1:**
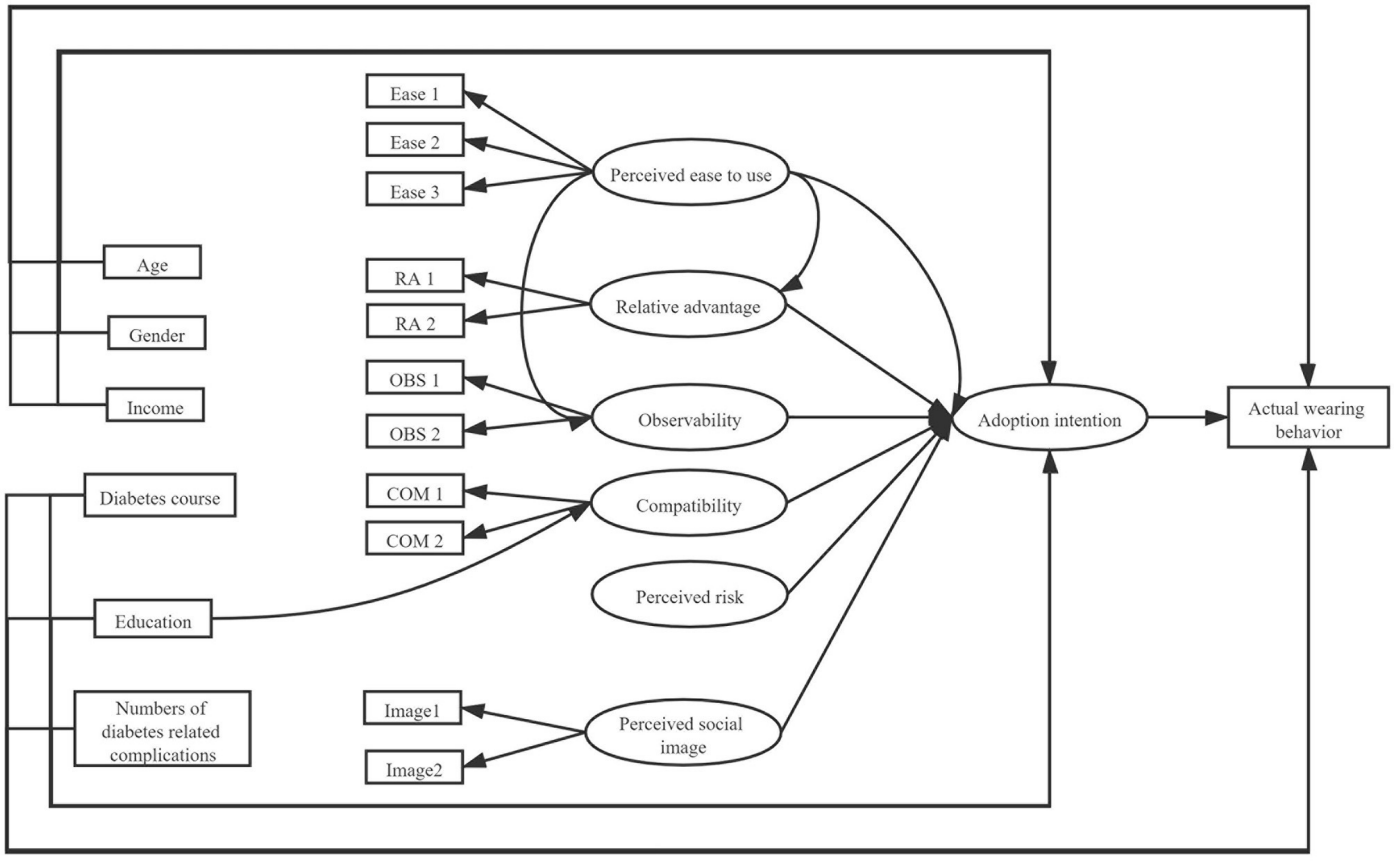
Proposed hypothesis in the current study.

Apart from the above factors, sociodemographic factors were also introduced to our hypotheses and the overall research model was reflected in Figure 1. The hypotheses are as follows:

H1: Perceived ease to use could directly influence elderly type 2 diabetic patients' intention to wearable activity trackers.
H2: Perceived ease to use could influence perceived relative advantages and then influence elderly type 2 diabetic patients' intention to wearable activity trackers.
H3: Compatibility of wearable activity trackers could directly influence type 2 diabetic patients' intention wearable activity trackers.
H4: Observability of wearable activity trackers could directly influence type 2 diabetic patients' intention wearable activity trackers.
H5: Perceived ease to use could directly influence observability and then influence elderly type 2 diabetic patients' intention to wearable activity trackers.
H6: Perceived risk could directly influence elderly type 2 diabetic patients' intention to wearable activity trackers.
H7: Education level of patients could directly influence their intention to wearable activity tracker and directly influence their actual wearing behavior.
H8: Education level of patients could influence perceived ease to use and then influence their intention to wearable activity tracker and finally influence their actual wearing behavior.
H9: Education level of patients could influence compatibility and then influence their intention to wearable activity tracker and finally influence their actual wearing behavior.
H10: Age of patients could directly influence their intention to wearable activity tracker and directly influence their actual wearing behavior.
H11: Gender of patients could directly influence their intention to wearable activity tracker and directly influence their actual wearing behavior.
H12: Diabetes course of patients could directly influence their intention to wearable activity tracker and directly influence their actual wearing behavior.
H13: Numbers of diabetes related complications could directly influence their intention to wearable activity tracker and directly influence their actual wearing behavior.
H14: Patients' intention to wearable activity tracker directly influences their actual wearing behavior.

### Aims

Therefore, this study aims to (1) explore the predicting factors of Chinese elderly type 2 diabetic patients' adoption intention to wearable activity trackers; (2) explore the influencing factors of actual wearing behavior. As wearable activity trackers only emerged in recent years and remain an innovation technology, this study will use Rogers' DOI to analyze factors influencing patients' adoption intention. To our knowledge, this study is the first to explore the predicting factors of adoption intention and actual behavior to wearable activity trackers among Chinese elderly patients with type 2 diabetes.

## Methods

### Study Settings and Sample

This study was an additional research of a randomized controlled trial supported by the National Natural Science Foundation of China. Cluster sampling was used to recruit participants. With the help of medical workers in the community health center, we noticed all the people meeting inclusion and exclusion criteria. Those who agreed to participate were included in this study. Patients with type 2 diabetes were recruited from Beijing, China, if they were aged between 18 and 75 years; permanent residents in their District; and did not take any psychiatric medications before recruitment. Subjects were excluded if they had type 1 diabetes, gestational diabetes, or secondary diabetes; severe complications of the heart, brain, kidney, eye, foot, and nervous system; mental disorders; tumor; or they were currently participating in similar studies.

This study included 819 type 2 diabetes patients. According to the requirements of the structural equipment model proposed by Preacherji and Coffman (33), the degree of freedom of the structural equation model was 117. When α = 0.05, β = 0.1 and RMSEA = 0.05, the required sample size was 182, so the sample size was totally enough.

This study was approved by the Institutional Review Board of Peking University. Informed consent was obtained from all participants before they were enrolled in the study.

### Device

All subjects were provided with wearable activity trackers (Mambo HR, Lifesense Co.) at baseline and instructed how to use them including how to wear and check related data. The wearable activity tracker has three main functions. First, it could track the steps, miles and calories consumption, heart rate, and sleep status of the users automatically. Second, it could generate the related data for users to check. So, users could view all the related data of themselves. Third, all the data would be uploaded into the exclusive cloud for researchers. As long as the users wear the tracker, the data would be generated and uploaded.

### Questionnaire

The questionnaire consists of two parts including the Diffusion of Innovation Scale and socio-demographic characteristics questionnaire. Diffusion of Innovation Scale was designed according to the essential factors of DOI including relative advantage, observability, compatibility, perceived complexity, perceived social image, and perceived risk (29, 31, 34–36). As is seen in Table 1, this scale was also tested by confirmatory factor analysis. The socio-demographic characteristics questionnaire was designed to collect patients' basic information including gender, age, education level, income, marital status, and so on.

**Table 1:** Dimensions of diffusion of innovation.

**Dimensions of DOI**	**Descriptions**	**Number of items**	**Example**	**Cronbach's alpha**
Relative advantage	Relative advantage reflected the degree to which wearable activity trackers were perceived useful by type 2 diabetes patients.	2	The wearable activity tracker can urge me to exercise.	0.725
Observability	Observability referred to whether the benefits of wearable activity tracker were easily observed and visible.	2	After using the wearable activity tracker for a period of time, I can quickly understand the advantages and convenience of the wearable activity tracker.	0.752
Compatibility	Compatibility referred to whether wearable activity tracker was compatible with patients' values, beliefs, experiences, and needs.	2	How often do you shop online?	0.796
Perceived complexity	Perceived complexity reflected the efforts patients demonstrated when trying to adopt wearable activity trackers.	3	I can easily use the wearable activity tracker to monitor my movement	0.803
Trialability	Trialability referred to the degree to which an innovation could be tried by potential users.	/	/	/
Perceived social image	Wearable activity trackers had the function of accessories and users might adopt an innovation technology to reflect their social status and improve social image.	2	Wearing a wearable activity tracker makes me feel more fashionable	0.807
Perceived risk	Perceived risks have been established to be influential to user acceptance of technology.	1	Using the wearable activity tracker will affect my privacy.	0.981

### Actual Wearing Behavior

All the data generated during using the wearable activity tracker were uploaded into the exclusive cloud. Data would not be generated when the patient did not use it. So, the actual wearing days could be calculated and the number of actual wearing days was seen as outcome variable.

### Data Collection

In this study, data were collected through two approaches. On the one hand, after the patients have tried the wearable activity trackers for a week, their socio-demographic characteristics, intention to continue to use wearable activity trackers, and the possible influencing factors were collected by related questionnaire. The survey was conducted on the spot. All the patients with free wearable activity trackers were informed to the community health station closest to them at a certain time and trained investigators conducted one-on-one inquiries. Presents would be given to those who cooperated with the investigation. On the other hand, the data of actual wearing behavior were collected by the exclusive cloud directly. Finally, a total of 725 patients completed the questionnaire survey.

### Statistical Analysis

Data were entered with Epidata 3.1. Statistical analysis was performed with Stata/S.E. 14.1 and Mplus7. The normality test was performed on continuous data. Those following the normal distribution were reported as mean ± SD and others were described by median and interquartile range. Categorical data were reported in frequency and proportion.

We used a two-step approach to analyze the structural equation model including the measurement model and the structural model. A measurement model namely CFA was used to evaluate whether the scale was qualified before performing the structural model. Only when the validity and reliability of the scale were qualified, it was meaningful to perform the structural model. Otherwise, the persuasiveness of the results of the structural model would be greatly reduced. Composite reliability and Cronbach's alpha were used to measure the degree of reliability of each construct. Generally, the reliability is considered to be satisfactory when composite reliability is >0.6 and Cronbach's alpha is >0.7 (36). The structural model namely structural equation modeling (SEM) was conducted to examine the research hypotheses. In SEM, χ2, Comparative Fit Index (CFI), Tucker–Lewis Index (TLI), Standardized Root Mean Square Residual (SRMR), and Root Mean Square Error of Approximation (RMSEA) were used to evaluate model fitness. Currently, literature recommended a χ2/degree of freedom < 5 (37), CFI > 0.90 (37), TLI > 0.90 (38), RMSEA < 0.08 (39) and SRMR < 0.08 (40). Full Information Maximum Likelihood estimation was used to deal with missing data.

## Results

### Participants' Characteristics

Table 2 shows the demographic characteristics of study participants. A total of 725 patients were included in this study, 342 male patients and 383 female patients. The majority of patients were the elderly, with a mean age of 60.3 ± 7.6 years old. The educational level of recruited patients was generally lower, with only 17.9% of patients receiving a college education and 25.6% receiving a high school education. The vast majority of participants (94%) were married and 40.4% of patients had an average monthly family income of 4,001 RMB and above. The mean duration of having type 2 diabetes in this study was 5.8 ± 3.5 years and the majority of patients (66.9%) had no diabetes-related complications.

**Table 2:** Demographic characteristics of the participants.

	***N***	**%**
**Gender**		
Male	342	47.2
Female	383	52.8
Age (years old, mean/SD)	60.3	7.6
**Education**		
Primary and below	83	11.5
Junior school	325	45.0
High school	185	25.6
College and above	129	17.9
**Marital status**		
Unmarried	2	0.3
Married	675	94.0
Divorced	10	1.4
Widowed	31	4.3
**Family income per person per month (RMB)**		
<3,000	206	28.7
3,000–4,000	221	30.9
4,001–5,000	100	14.0
>5,001	189	26.4
Duration of diabetes (year, mean/SD)	5.8	3.5
**Number of complications**		
None	485	66.9
1	162	22.3
2	49	6.8
≥3	29	4.0
Total	725	100

### Measurement Model Assessment

The results of CFA were presented in Tables 3, 4 (χ^2^/df = 2.9, CFI = 0.975, TLI = 0.961, RMSEA = 0.052, SRMR = 0.030). Results in Table 3 showed that this scale had good reliability. Validity was measured by average variance extracted (AVE) and the correlation coefficient matrix of latent variables (Tables 3, 4). The values of AVE in this study were all above the standard of 0.5 and the square root of AVE of each construct was higher than its correlation with other constructs, indicating a good validity of the scale (36, 41). Therefore, based on the above results, this measurement model was suitable for structural model testing.

**Table 3:** Confirmatory factor analysis results of the measurement model.

**Constructs**	**Items**	**Factor loading**	**CR**	**AVE**
Relative advantage			0.725	0.569
	RA1	0.826		
	RA2	0.701		
Ease to use			0.812	0.593
	Ease1	0.729		
	Ease2	0.911		
	Ease3	0.683		
Perceived observability			0.755	0.607
	OBS1	0.772		
	OBS2	0.785		
Perceived compatibility			0.815	0.693
	COM1	0.853		
	COM2	0.797		
Social image			0.807	0.678
	Image1	0.822		
	Image2	0.823		
Intention			0.762	0.619
	INT1	0.783		
	INT2	0.789		
**Total**			0.911	0.622

*RA, Relative advantage; Ease, Ease to use; OBS, Observability; COM, Compatibility; Image, Perceived social image; INT, Adoption intention; CR, Composite reliability; AVE, Average variance extracted*.

**Table 4:** Correlation coefficient matrix and square root of average variance extracted of latent variables.

	**RA**	**EASE**	**OBS**	**COM**	**Image**	**INT**
Relative advantage (RA)	0.754^a^					
Ease to use (Ease)	0.645^***^	0.770^a^				
Perceived observability (OBS)	0.737^***^	0.750^***^	0.779^a^			
Perceived compatibility (COM)	0.030	0.193^***^	0.112^*^	0.832^a^		
Social image (Image)	0.228^***^	0.094^*^	0.296^***^	−0.022	0.823^a^	
Intention (INT)	0.716^***^	0.609^***^	0.855^***^	< -0.001	0.247^***^	0.787^a^

* *P < 0.05*;

*** *P < 0.001*.

a *Square root of average variance extracted of latent variables*.

### Structural Model for Adoption Intention

**Figure 2:**
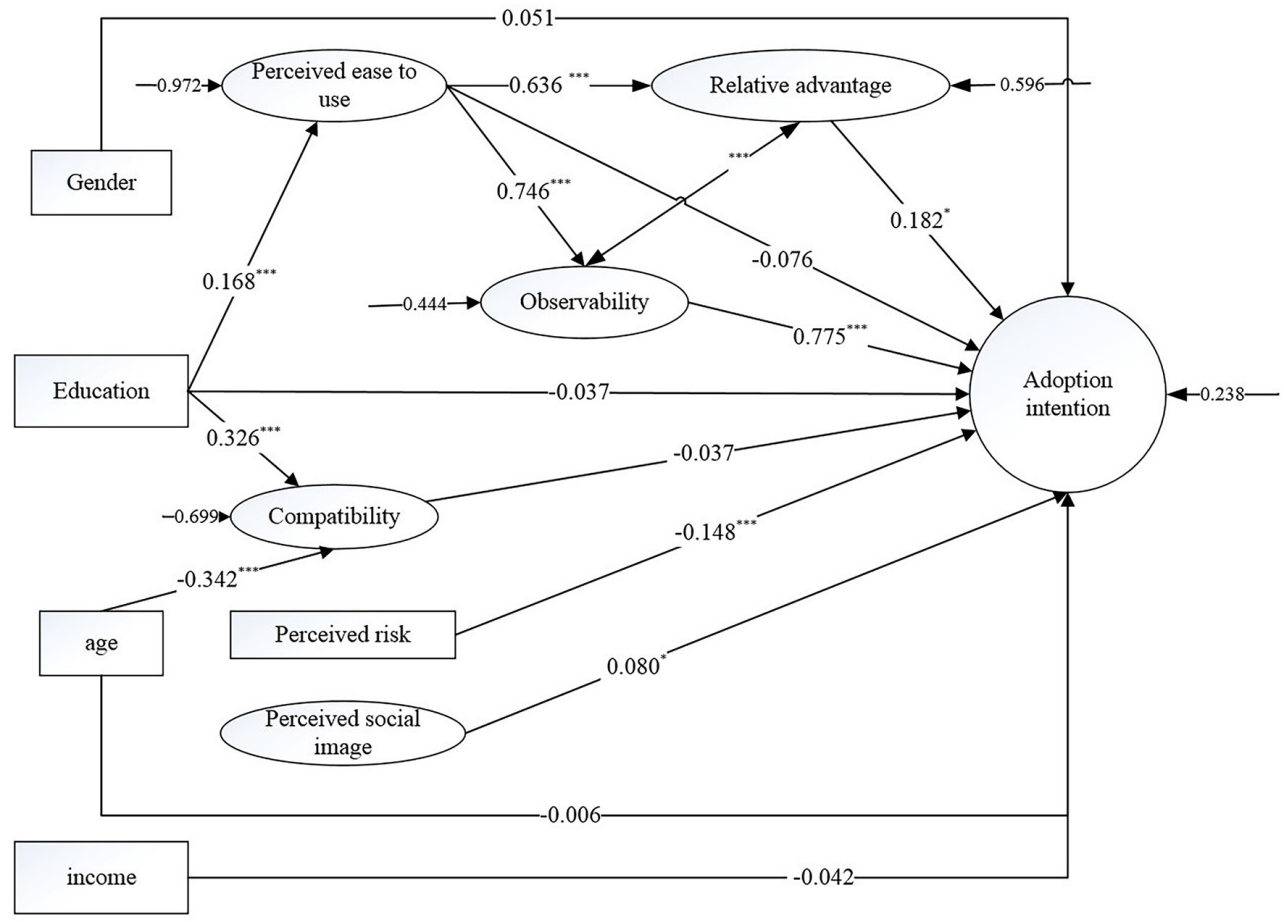
Results of structural equation model for adoption intention.

The model fit indices were χ^2^/df = 3.5, CFI = 0.931, TLI = 0.914, RMSEA = 0.060, SRMR = 0.068, which indicated that the proposed model could properly represent the hypothesized relationships. Structural equation model results were presented in Figure 2. As only one item was used to measure perceived risk, perceived risk was processed as an observed variable.

In the model, gender, education, and age had no significant direct influence on patients' adoption intention. However, education had a significant indirect effect through perceived ease to use on patients' adoption intention (indirect effect size = 0.104, *P* < 0.001). Although age and education had a significant influence on compatibility, the effect was insignificant on patients' adoption intention. Furthermore, the total effect of education and age on adoption intention were insignificant also.

Perceived ease to use had no direct significant influence on adoption intention. However, it had a moderating effect through relative advantage (indirect effect size = 0.116, *P* = 0.015) and observability (indirect effect size = 0.578, *P* < 0.001). Furthermore, the total effect of perceived ease to use was 0.618 (*P* < 0.001). Observability had a direct effect of 0.775 (*P* < 0.001) and relative advantage had a direct effect of 0.182 (*P* = 0.014). Compatibility had an insignificant influence on the adoption intention of patients (*P* = 0.409) and perceived risk had a negative impact of −0.148 (*P* < 0.001). Perceived social image could positively affect the adoption intention of patients (β = 0.080, *P* = 0.039).

### Structural Model for Actual Wearing Behavior

**Figure 3:**
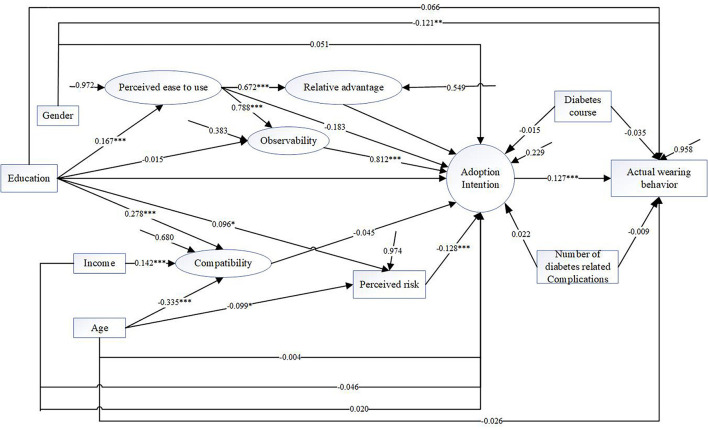
Results of structural equation model for actual wearing behavior.

The model for actual wearing behavior is shown in Figure 3. The model fit indices were χ^2^/df = 359.308/118.000 = 3.045, CFI = 0.935, TLI = 0.914, RMSEA = 0.054, SRMR = 0.052. Social image had so minor effect, so it didn't enter the model. Results showed that adoption intention influenced the actual wearing behavior directly and significantly (direct effect size = 0.127, *P* = 0.003). Among the variables in DOI, the primary influencing factor was observability (indirect effect size = 0.103, *P* = 0.005), which is consistent with the adoption intention. Moreover, the perceived ease (indirect effect size = 0.085, *P* = 0.004) and relevant advantage (indirect effect size = 0.041, *P* = 0.009) also had indirect significant effect on actual wearing behavior.

The influence of sociodemographic variables on behavior is different from that of adoption intention. Gender had a significant direct effect on the actual wearing behavior, the wearing days of men were significantly higher than those of women (direct effect size = −0.121, *P* = 0.003). Education had a significant indirect effect on behavior through 2 paths: education-“perceived ease”-“relevant advantages”-“adoption intention”-“actual wearing behavior” (indirect effect size = 0.014, *P* = 0.020) and education-“perceived ease”-“observability”-“adoption intention”-“actual wearing behavior” (indirect effect size = 0.005, *P* = 0.029).

## Discussion

### Principal Findings

With the introduction of perceived social image and perceived risk to classic diffusion of innovation theory, this study aims to analyze the influencing factors of Chinese diabetic patients' adoption intention to wearable activity trackers and their actual wearing behavior. This study is important in the way that future interventions could well use the influencing factors identified in this study to promote the usage of wearables by type 2 diabetic patients and further improve their blood glucose control.

Although currently there are some studies exploring the perspectives and the adoption intention to wearable devices by users, most of them were conducted in healthy and young populations and the sample size was relatively small (17, 34, 42). But with the progress of age, how to use this kind of technology in their life is much more important. Furthermore, some studies only let participants try wearable devices for a few days (such as 3 days) and some only let participants imagine their use of wearables rather than actually experiencing them (30, 32, 34). Only through a short-term of try-on, participants could not fully understand the functions of wearables or fully encounter possible problems. Therefore, in this study, we included 725 elderly diabetic patients and conducted questionnaires after a week's try-on to explore potential influencing factors and completed a 3-month follow-up.

Adoption intention is the main and direct influencing factor of the actual wearing behavior. Exploring the influencing factors of adoption intention could help understand the complete behavior progress of type 2 diabetic patients. Different from some related studies conducted in elderly populations where relative advantage and perceived ease to use often exerted the most influence on users' intention (30, 32, 34), we found that observability was the primary predicting factors of patients' adoption intention in this study and perceived ease to use was the second. Furthermore, interestingly, perceived ease to use did not have a direct effect on adoption intention but exerted an influence through perceived relative advantage and observability. This result was similar to Ma's and Li's studies (24, 32). In these two studies examining smartphone technologies and wearable technologies in elderly population, they also found that perceived ease to use did not directly affect intention, but exerted an influence through relative advantage. This might be because wearable activity trackers are already easy to use and users do not need to make substantial efforts to learn it. Furthermore, in this study, perceived relative advantage was the third influential factor of adoption intention and only had an effect size of 0.182. The diminishing effect of relative advantage and ease to use corroborated studies by Deng et al. and Hu et al. (36, 43). In these two mHealth and telemedicine studies, they also found a diminishing effect of perceived ease to use and perceived relative advantage. One possible explanation might be that compared with users several years ago, the current users are more technology savvier, and modern products such as wearable activity trackers are less complicated to learn and it is hard for them to encounter a novel advantage. Moreover, in other adoption intention studies, observability was seldom discussed. However, in this study, observability had the most influence on both adoption intention and actual wearing behavior. This result might indicate that in the special population of elderly diabetic patients if they could observe a product' benefits more easily, they are more likely to adopt it. Therefore, future interventions might need to emphasize more on the easily observed characteristics of new products and clearly explain potential benefits to users to facilitate the observation process. And observability not only did effect on adopting intention but also the actual wearing behavior indirectly. The reason may be that observability could stimulate people's enthusiasm to manage themselves.

The results in terms of perceived risk and perceived social image were similar to those in other studies (26, 30). Cimperman et al. found that the trust in technology the elderly users had a strong positive influence on their adoption intention to home telehealth services and Takemoto el al. found that older adults might have different definitions of risk regarding data control compared with younger populations (30, 44). Generally, elderly people tended to be more conservative than younger generations and they might avoid decision-making and refuse to try new products to reduce possible risks (45). Therefore, in future studies, when communicating with participants, it is important to eliminate their possible doubts and worries in terms of product usage. The study results also revealed a positive influence on perceived social image. Puri et al. studied older adults' adoption intention to wearable activity trackers and they have also found a positive influence of activity trackers' aesthetics aspect (23). Furthermore, Venkatesh et al. explored the influencing factors of personal laptop buying behavior and the result also indicated that the most important factor stimulating users' behavior was that personal laptop could improve their social image (26). Wearable activity trackers in this study had aesthetic features and accessory functions. Therefore, patients might think that the wearing of wearables could improve their image and social status, which could, in turn, promote adoption behavior.

Socio-demographic characteristics including gender, age, and education did not exert a significant direct influence on adoption intention. But gender had a significant direct effect on actual wearing behavior. One possible explanation might also be that the wearable activity tracker itself did not incorporate many complicated technologies and did not need users to make substantial efforts to learn it. Therefore, a relatively easier operation system is not demanding on users' age or educational background. But it seems that men are always more interested in electronic products than women, so they tended to use the wearable tracker more frequently. Furthermore, as age did not impede patients' adoption intention to wearable activity trackers, it is possible to promote wearables in this population to help them control blood glucose.

### Implications and Limitations

Wearable activity trackers are proven to be effective in lowering diabetes patients' blood glucose in China (17), so it is meaningful to let more diabetes patients accept this technology and help them manage their blood glucose. China is a developing country with high developing speed, so wearable activity trackers are accessible to patients with diabetes. Using mobile health such as wearable activity trackers to manage blood glucose is a promising way because of the large number of diabetes patients in China. As a new thing, identifying the influencing factor of adoption intention and actual wearing behavior could help wearable activity trackers' diffusion. Especially, with the aging progress, applying the new technologies into their life is much more important.

This study identified the influencing factors of adoption intention of type 2 diabetic patients to wearable activity trackers and showed the actual wearing behavior in 3-month. The results indicated that adoption intention was the main influencing factor of actual wearing behavior and observability was the primary influencing factor of adoption intention. Besides, gender also influenced their actual wearing behavior, and the influences of relative advantage and perceived ease to use in terms of simple technologies such as wearable activity trackers are diminishing. Therefore, in order to encourage diabetic patients to use wearable activity trackers to manage their blood glucose, it is significant to improve their adoption intention first. As mentioned above, observability should be paid more attention. When designing or introducing new technologies for diabetic patients, it is a good strategy to make users' progress and action visible. Take the trackers in this study as an example. Patients could see their health data through the tracker explicitly. Although the relative advantage and perceived ease to use showed limited influence in this study, it does not mean they are insignificant. It just indicated that wearable activity trackers were so easy for them to use. Compared with complicated devices, the simple ones may still have advantages. Furthermore, patients' doubts should also be reduced as much as possible, such as protecting their privacy well, avoiding bundled consumption items for them, and so on. It is helpful to decrease their perceived risk and then improve their adoption intention. For women, they may need more patience to encourage them to have a try because they tend to be more likely to refuse use. Future interventions could try to synthesis these suggestions together to generate more aesthetical products to help them manage blood glucose.

This study also has some limitations. First, the wearable activity trackers were freely distributed to patients. Therefore, patients' actual buying behavior in natural settings could not be measured. Second, patients recruited in this study had fewer complications and shorter duration of diabetes. Therefore, their state of illness might be less serious and more suitable for physical activities. The extrapolation of the study result to a population with more serious illness should be cautious. Third, patients' perceived risk was measured with only one item and this construct was treated as an exogenous variable in the structural equation model. Therefore, this might introduce some biases.

## Conclusion

This study used the diffusion of innovation theory as a tool and explored the predicting factors of Chinese elderly type 2 diabetic patients' adoption intention to wearable activity trackers and tracked their actual wearing behavior in 3-month. The results indicated that adoption intention was the main direct influencing factor of adoption behavior. And observability, perceived ease to use, relative advantage, perceived social image had a positive influence on patients' adoption intention while perceived risk exerted a negative influence. Especially, observability also exerted an indirect influence on actual wearing behavior. Controlled variables including gender, age, and education did not exhibit a significant influence on adoption intention but gender had a direct significant effect on wearing behavior. These findings are conducive to future research on wearable activity trackers' adoption studies.

## Data Availability Statement

The raw data supporting the conclusions of this article will be made available by the authors, without undue reservation.

## Ethics Statement

The studies involving human participants were reviewed and approved by the Institutional Review Board of Peking University. The patients/participants provided their written informed consent to participate in this study.

## Author Contributions

XS and XF designed this study. PC and YS completed the manuscript. ZL processed some of the data. EF guided the investigation. All authors contributed to the article and approved the submitted version.

## Funding

This work was funded by the National Natural Science Foundation of China (Grant Nos. 72174008 and 71673009).

## Conflict of Interest

The authors declare that the research was conducted in the absence of any commercial or financial relationships that could be construed as a potential conflict of interest.

## Publisher's Note

All claims expressed in this article are solely those of the authors and do not necessarily represent those of their affiliated organizations, or those of the publisher, the editors and the reviewers. Any product that may be evaluated in this article, or claim that may be made by its manufacturer, is not guaranteed or endorsed by the publisher.
